# Draft Genome Sequences of Four Alkaliphilic Bacteria Belonging to the *Anaerobacillus* Genus

**DOI:** 10.1128/genomeA.01493-16

**Published:** 2017-01-19

**Authors:** Naji M. Bassil, Jonathan R. Lloyd

**Affiliations:** Research Centre for Radwaste and Decommissioning and Williamson Research Centre for Molecular Environmental Science, School of Earth and Environmental Sciences, University of Manchester, Manchester, United Kingdom

## Abstract

The draft genomes of the alkaliphilic, anaerobic bacteria, *Anaerobacillus arseniciselenatis*, *A. alkalidiazotrophicus*, and *A. alkalilacustris*, and a novel closely related isolate of the *Anaerobacillus* genus are reported here. These assembled genomes will help identify, at the molecular level, the phenotypic differences between the species of this poorly characterized genus.

## GENOME ANNOUNCEMENT

The species *Bacillus arseniciselenatis* (1), *B. alkalidiazotrophicus* (2), and *B. macyae* (3) were transferred in 2009 to the newly established genus *Anaerobacillus* as *A. arseniciselenatis* comb. nov., *A. alkalidiazotrophicus* comb. nov., and *A. macyae* comb. nov., respectively (4). Furthermore, *A. alkalilacustris* was also added to the genus *Anaerobacillus* (4). Species belonging to this genus are anaerobic or aerotolerant, Gram-positive rods that grow under obligate or moderately alkaliphilic and halophilic conditions, through fermentative or anaerobic respiration (4). The genome of the moderately alkalitolerant *A. macyae* DSM 16346 was reported recently and has the GenBank accession number LELK00000000 (5); however, genome sequences have not been reported to date for the obligate alkaliphilic bacteria assigned to this genus.

Isosaccharinic acid (ISA) is a polyhydroxycarboxylic acid that is important in the geologic disposal of radioactive waste, as it is the product of the abiotic, alkaliphilic hydrolysis of cellulose (6) and has the potential to mobilize radionuclides in the geosphere (7). A novel bacterium, *Anaerobacillus* sp. NB2006, whose 16S rRNA gene sequence aligned with species of the genus *Anaerobacillus*, was isolated in a study of the microbial degradation of ISA at high pH (8).

Here, we report the draft genomes of the obligate alkaliphiles *A. arseniciselenatis* DSM 15340, *A. alkalidiazotrophicus* DSM 22531, and *A. alkalilacustris* DSM 18345, and the novel ISA-degrading bacterium *Anaerobacillus* sp. NB2006. Cells were harvested at the late log phase by centrifugation at 4,000 × *g* for 10 min, and gDNA was extracted using the All-in-One purification kit (Norgen), following the protocol for Gram-positive bacteria. The DNA from each bacterium was sheared to 200 to 1,000 bp using NEBNext dsDNA Fragmentase (New England BioLabs), and barcoded libraries were prepared using the NEBNext Ultra DNA library prep kit for Illumina (New England BioLabs). Whole-genome sequencing of the pooled libraries was performed on a MiSeq platform (Illumina, San Diego, CA, USA), using V2 reagents to produce 250-bp paired-end reads. The barcode-separated raw reads were quality trimmed, and the sequencing adaptors were removed using Trimmomatic version 0.36 (9); then the PhiX sequences were removed and the sequence quality was checked using FaQCs version 1.34 (10). Overlapping reads were then joined into longer sequences using FLASh version 1.2.11 (11), and the resulting joined and unjoined sequences were *de novo* assembled using A5-miseq version 2015 (12), SOAPdenovo version 2.04 (13), ABySS version 2.0 (14), and SPAdes version 3.9 (15). The resulting assemblies were combined into a consensus assembly for each genome using CISA (contigs less than 1 kb were removed) (16), and this was later aligned to the previously published *A. macyae* DSM 16346 genome (GenBank accession no. LELK01000000) and scaffolded using Scaffold_Builder version 2.2 (17). Annotation of the genomes was performed using the NCBI Prokaryotic Genome Annotation Pipeline after sequence submission to GenBank. Although the G+C mol% of the four genomes were slightly lower than the values acquired by high-pressure liquid chromatography, they were well within the values expected for genomes from the related genus *Bacillus* (Table 1).

**TABLE 1:** Genome features and GenBank accession numbers of four *Anaerobacillus* spp.

Strain	Genome size (Mb)	No. of contigs	Longest contig (bp)	*N*_50_ contig length (bp)	No. of genes	Accession no.
*A. arseniciselenatis* DSM 15340	3.95	58	942,622	220,456	3,762	MLQQ00000000
*A. alkalidiazotrophicus* DSM 22531	4.61	36	1,531,730	641,971	4,263	MLQS00000000
*A. alkalilacustris* DSM 18345	4.05	50	818,671	262,426	3,798	MLQR00000000
*Anaerobacillus* sp. NB2006	4.95	211	256,216	56,969	4,984	LQXD00000000

### Accession number(s).

The assembled and annotated genome sequences were deposited in GenBank under the accession numbers listed in Table 1.
